# Brief Report: Clinical Outcomes by Infusion Timing of Immune Checkpoint Inhibitors in Patients With Locally Advanced NSCLC

**DOI:** 10.1016/j.jtocrr.2024.100659

**Published:** 2024-03-05

**Authors:** Tsuyoshi Hirata, Yuji Uehara, Taiki Hakozaki, Takayuki Kobayashi, Yuto Terashima, Kageaki Watanabe, Makiko Yomota, Yukio Hosomi

**Affiliations:** a Department of Thoracic Oncology and Respiratory Medicine, Tokyo Metropolitan Cancer and Infectious Diseases Center, Komagome Hospital, Tokyo, Japan; b Department of Precision Cancer Medicine, Center for Innovative Cancer Treatment, Tokyo Medical and Dental University, Tokyo, Japan; c Department of Pulmonary Medicine and Oncology, Graduate School of Medicine, Nippon Medical School, Tokyo, Japan

**Keywords:** Immunotherapy, Lung cancer, Durvalumab, Circadian rhythms, Chronotherapy

## Abstract

**Introduction:**

Previous studies reported an association between immune checkpoint inhibitor infusion timing and the treatment effect in metastatic NSCLC. The present study assessed the association between durvalumab infusion timing and survival outcomes in patients with locally advanced NSCLC.

**Methods:**

Patients receiving durvalumab after chemoradiotherapy for locally advanced NSCLC at a single institution were retrospectively analyzed, and the association of the proportion of durvalumab infusions greater than or equal to 20% versus less than 20% after 3 PM with progression-free survival (PFS) and overall survival was assessed.

**Results:**

A total of 82 patients were included, with a median age of 69 years (interquartile range, 62–74 years); of these, 67 patients (82%) were of male sex, and 78 patients (95%) had a history of smoking. The median number of durvalumab infusions per patient was 16 (interquartile range, 8–24). Patients with at least 20% of their durvalumab infusions after 3 PM (n = 12/82, 15%) had a significantly shorter PFS than those who did not (median: 7.4 mo versus not available [NA]; hazard ratio [HR], 2.43; 95% confidence interval [CI]: 1.11–5.34, *p* = 0.027), whereas overall survival was shorter among the former compared with the latter group (median: 22.4 versus NA; HR, 1.80; 95% CI: 0.73–4.42, *p* = 0.20). In addition, both backward stepwise multivariable analysis and propensity score–matching analysis revealed that receiving at least 20% of durvalumab infusions after 3 PM was significantly associated with worse PFS (HR, 2.54; 95% CI: 1.03–5.67, *p* = 0.047; and HR, 4.64; 95% CI: 1.95–11.04; *p* < 0.001, respectively).

**Conclusions:**

The time of day of durvalumab infusions may impact survival outcomes in patients with locally advanced NSCLC.

## Introduction

The addition of consolidative durvalumab therapy to concurrent chemoradiation (cCRT) has improved survival outcomes in patients with locally advanced NSCLC. However, our understanding of the factors contributing to this therapy’s efficacy is limited.

Circadian genetic and hormonal signals strongly influence a wide variety of systemic immune functions, including the activation of innate and adaptive immunity.^1^ At the tumor level, circadian clock genes are closely associated with immune signaling, activation, and immunophenotype in various cancer types.^2^^,^^3^ In addition, vaccine-induced immunity diminishes with evening administration.^3^, ^4^, ^5^

The association between the time of day of immune checkpoint inhibitor (ICI) infusions and their efficacy was first reported in patients with metastatic melanoma.^6^ This study observed that overall survival (OS) was significantly shorter among patients receiving at least 20% of their ICI infusions after 4:30 PM than those receiving less than 20% of these treatments after 4:30 PM (*P* = 0.025).^6^ Similar studies in metastatic NSCLC and pantumor meta-analysis also found that patients receiving late time-of-day infusions had significantly shorter progression-free survival (PFS) than those receiving early time-of-day infusions.^10^, ^7^, ^8^, ^9^ However, the association between the time of day of ICI infusions and clinical outcomes in patients with locally advanced NSCLC remains unknown.

Here, we evaluated the association between patterns in the timing of durvalumab infusions and survival outcomes in patients with locally advanced NSCLC. Backward stepwise multivariable regression and propensity score-matching (PSM) were performed to minimize bias.

## Materials and Methods

### Study Design

Patients with newly diagnosed or locally recurrent unresectable, locally advanced NSCLC who completed platinum-based cCRT and received durvalumab consolidation therapy between January 2018 and May 2022 at the Department of Thoracic Oncology and Respiratory Medicine at Tokyo Metropolitan Cancer and Infectious Diseases Center of Komagome Hospital (Tokyo, Japan) were identified. Patients who received fewer than four durvalumab infusions were excluded from these analyses. Baseline demographic, clinical, and treatment data were collected from the patients’ electronic medical records. NSCLC histologic diagnosis and disease stage were classified according to the WHO criteria and the American Joint Committee on Cancer and International Union Against Cancer (version 8) TNM criteria, respectively. Programmed death-ligand 1 (PD-L1) expression was immunohistochemically assessed in tissue biopsy samples (DAKO 22C3; Agilent Technologies, Santa Clara, CA). Genomic molecular profiles of the patients’ tumors were analyzed with next-generation sequencing and single-gene sequencing using tumor tissue. This study was approved by the institutional review board of the Tokyo Metropolitan Cancer and Infectious Diseases Center at Komagome Hospital (number 2725). Because this study was retrospective, the requirement for individual patient consent for participation was waived.

### Outcomes

PFS was defined as the duration from the first administration of durvalumab to any disease progression or death. Patients were censored at their first progression event. OS was defined as the first administration of durvalumab from the start of radiotherapy until death from any cause. Patients were censored at the date of their last follow-up.

### Statistical Analysis

The relationship between the percentage of patients receiving durvalumab infusions after 3 PM and their survival outcomes was assessed. On the basis of previous research, a threshold of infusions greater than or equal to 20% versus less than 20% received after the 3-PM cutoff time (the onset of durvalumab infusion) was adopted for the analysis.^6^, ^7^, ^8^ Although most previous studies used 4:30 PM as the cutoff time (Supplementary Table 1),^10^ none of the patients in our cohort met this criterion because of the clinic's closing time. Consequently, we adjusted the cutoff time to 3 PM.

The association between the percentage of durvalumab infusions received after 3 PM, and both PFS and OS was first estimated using a univariable Cox proportional hazard model, then a backward stepwise multivariable Cox regression model incorporating other clinically relevant variables (age, sex, performance status, smoking status, histologic subtypes, PD-L1 expression, lung immune prognostic index groups,^11^ stage, and number of durvalumab infusions). For the sensitivity analysis, to account for potential confounding factors when comparing survival outcomes between patients receiving at least 20% of their durvalumab after 3 PM and those receiving less, all patients were matched using PSM. The matching was on the basis of variables such as age, sex, smoking status, histologic subtypes, PD-L1 expression, lung immune prognostic index groups, stage, and the number of durvalumab infusions. Patients receiving at least 20% of their durvalumab after 3 PM and those receiving were matched at a ratio of 5:1 using the greedy matching method with a caliper of 0.2.^12^ In addition, we conducted a sensitivity analysis using 2:30 PM as an alternative cutoff time. Descriptive statistics were used for patient and tumor characteristics. A *p* value of less than 0.05 was considered statistically significant. All statistical analyses were performed using R version 4.1.1 (R Core Team, Vienna, Austria).

## Results

We identified 82 consecutive patients with locally advanced NSCLC treated with definitive-intent cCRT and durvalumab. Their median age was 69 years, with an interquartile range (IQR) of 62 to 74 years; 67 patients (82%) were of the male sex; and 36 patients (46%) had an Eastern Cooperative Oncology Group performance status of 0 (Table 1). There were 78 patients (95%) who were current or former smokers, whereas 38 patients (46%) had adenocarcinoma histology. PD-L1 expression was less than 1% in 18 patients (22%), 1% to 49% in 18 patients (22%), 50% or more in 23 patients (28%), and unavailable in 23 patients (28%). Stage IIIB or IIIC disease was found in 43 patients (52%). The median number of durvalumab infusions per patient was 16 (IQR: 8–24). Of all patients, 12 patients (15%) received at least 20% and 70 (85%) received less than 20% durvalumab infusions after 3 PM. The number of durvalumab infusions per patient was higher in the early infusion group than in the late infusion group (median: 17 versus 10). Otherwise, no significant imbalance was observed in the patient, disease, or treatment characteristics, including adverse events leading to permanent discontinuation of durvalumab.

**Table 1:** Patient Characteristics

Characteristics	Whole Population (N = 82)	Percentage Infusion After 3 PM <20% (n = 70)	Percentage infusion After 3 PM >20% (n = 12)	*p*
Age (median, IQR), n (%)	69 (62–74)	69 (64–74)	64 (55–68)	0.67
≤65 y	26 (32)	20 (29)	6 (50)	
>65 y	56 (68)	50 (71)	6 (50)	
Sex, n (%)				
Male	67 (82)	59 (84)	8 (67)	0.29
Female	15 (18)	11 (16)	4 (23)	
ECOG PS, n (%)				
0	38 (46)	31 (44)	7 (58)	0.81
1	41 (50)	37 (53)	4 (33)	
≥2	3 (4)	2 (3)	1 (8)	
Smoking status, n (%)				
Current or former	78 (95)	66 (94)	12 (100)	0.90
Nonsmoker	4 (5)	4 (6)	0	
Histologic subtypes, n (%)				
Adenocarcinoma	38 (46)	34 (49)	4 (33)	0.089
Squamous cell carcinoma	33 (40)	29 (41)	4 (33)	
NSCLC, NOS	11 (13)	7 (19)	4 (33)	
PD-L1 expression, n (%)				
<1%	18 (22)	15 (21)	3 (25)	0.81
1–49%	18 (22)	15 (21)	3 (25)	
≥50%	23 (28)	19 (27)	4 (33)	
Unknown	23 (28)	21 (30)	2 (17)	
AJCC eighth edition, overall stage, n (%)				
IIIA	39 (48)	32 (46)	7 (58)	0.67
IIIB	31 (38)	27 (39)	4 (33)	
IIIC	12 (15)	11 (16)	1 (8)	
T stage, n (%)				
T1/T0	13 (16)	10 (14)	3 (25)	0.51
T2	19 (23)	15 (21)	4 (33)	
T3	13 (16)	12 (17)	1 (8)	
T4	37 (45)	33 (47)	4 (33)	
N stage, n (%)				
N0	11 (13)	10 (14)	1 (8)	0.81
N1	8 (10)	6 (21)	2 (17)	
N2	41 (50)	35 (17)	6 (50)	
N3	22 (27)	19 (47)	3 (25)	
Driver alterations, n (%)				
EGFR mutation	2 (2)	2 (3)	0	0.25
ALK rearrangement	1 (1)	1 (1)	0	
ROS rearrangement	1 (1)	1 (1)	0	
KRAS mutation	1 (1)	1 (1)	0	
MET mutation	1 (1)	0	1 (8)	
Negative or unknown	76 (93)	65 (93)	11 (92)	
LIPI groups^a^				
Good	26 (31)	23 (33)	3 (25)	0.53
Intermediate	44 (54)	38 (54)	6 (50)	
Poor	12 (15)	9 (13)	3 (25)	
Number of durvalumab infusions (median, IQR)	16 (8–24)	17 (8–24)	10 (5–16)	0.018
Adverse events leading to permanent discontinuation of durvalumab				
Any	11 (13)	9 (13)	2 (17)	
Pneumonitis	9 (11)	8 (12)	1 (8)	1.0
Endocrinopathies	1 (1)	0	1 (8)	
Peripheral neuropathy	1 (1)	1 (1)	0 (0)	

AJCC, American Joint Committee on Cancer; ECOG, Eastern Cooperative Oncology Group; IQR, interquartile range; LDH, lactate dehydrogenase; LIPI, lung immune prognostic index; NLR, neutrophil-to-lymphocyte ratio; NOS, not otherwise specified; PS, performance status; PD-L1, programmed cell death ligand; ULN, upper limit normal.

a Good, intermediate, and poor LIPI is on the basis of the pretreatment laboratory values with the following cutoffs: (1) dNLR less than or equal to 3 and LDH less than or equal to ULN; (2) dNLR greater than 3 or LDH greater than ULN; and (3) dNLR greater than 3 and LDH greater than ULN, respectively.

**Figure 1:**
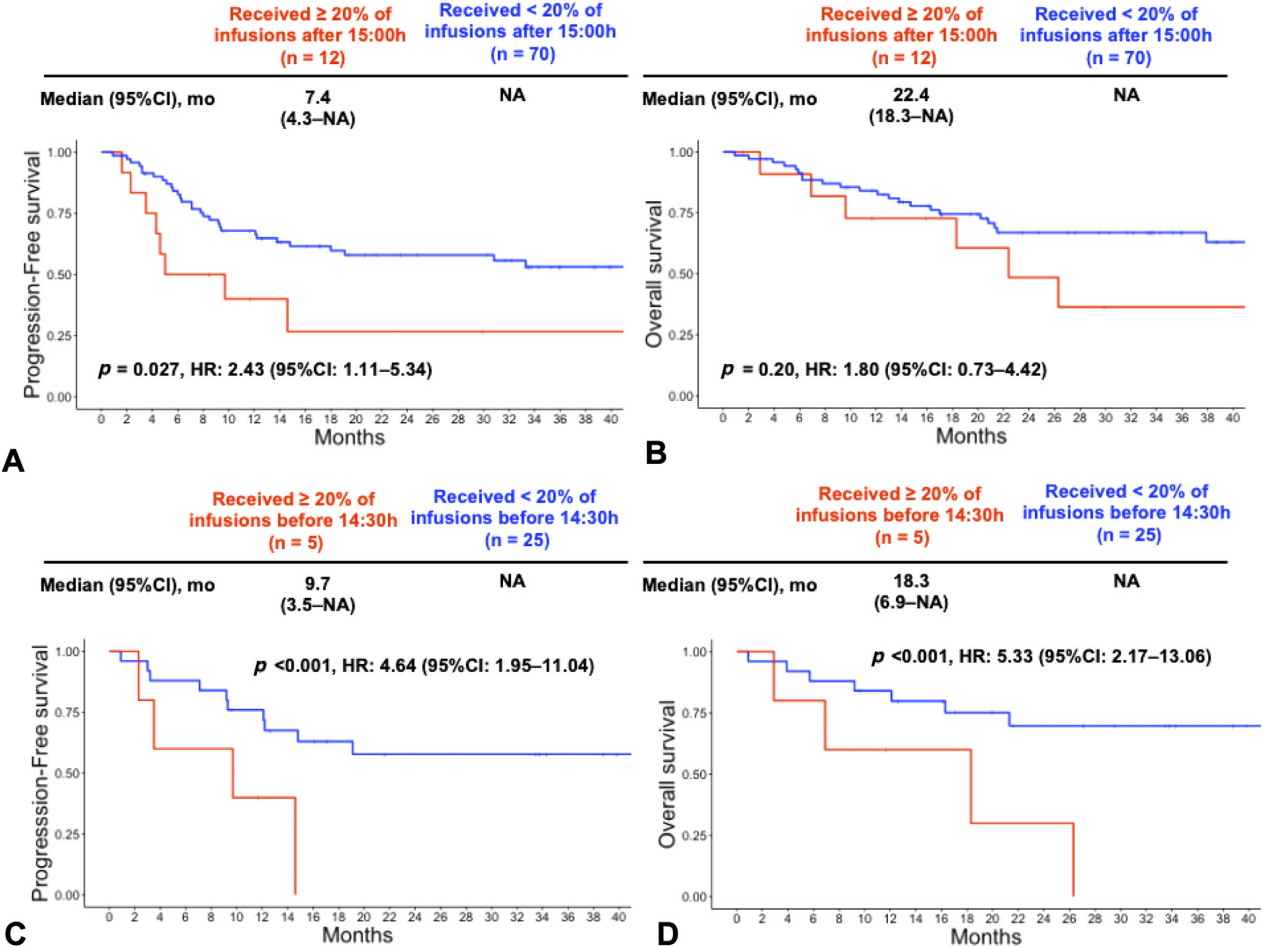
Kaplan-Meier curves according to a 20% threshold in the percentage of ICI infusions received after 3 PM. *(A)* Progression-free survival before PSM; *(B)* Overall survival before PSM. *(C)* Progression-free survival after PSM. *(D)* Overall survival after PSM. CI, confidence interval; HR, hazard ratio; NA, not available; PSM, propensity score-matching.

The median (IQR) follow-up period was 21 (12–36) months. Patients receiving at least 20% of their durvalumab infusions after 3 PM had a significantly shorter PFS than those who did not (median: 7.4 mo versus NA [not available]; hazard ratio [HR], 2.43; 95% confidence interval [CI]: 1.11–5.34; *p* = 0.027) (Fig. 1*A*). The OS was shorter in patients who received at least 20% of their durvalumab infusions after 3 PM than in those who did not (median: 22.4 mo versus NA; HR, 1.80; 95% CI: 0.73–4.42; *p* = 0.20) (Fig. 1*B*), although the difference was not statistically significant. On a backward stepwise multivariable analysis, receiving at least 20% of durvalumab infusions after 3 PM was associated with worse PFS (HR, 2.54; 95% CI: 1.03–5.67; *p* = 0.047) (Table 2), but there was no significant association with OS (HR, 1.28; 95% CI: 0.51–3.25; *p* = 0.60) (Table 2). Histology (squamous versus nonsquamous) and the number of durvalumab infusions were significantly associated with both PFS and OS (Supplementary Fig. 1).

**Table 2:** Univariable and Stepwise Multivariable Cox Model Analysis for Progression-Free Survival and Overall Survival (Cutoff: 3 PM)

Progression-Free Survival	Univariable Analysis			Multivariable Analysis^a^		
Variable	HR	95% CI	*p*	HR	95% CI	*p*
Age (≥65 vs. <65 years)	1.55	0.81–2.97	0.19			
Sex (male vs. female)	0.93	0.41–2.14	0.88			
ECOG PS (≥1 vs. 0)	1.48	0.77–2.84	0.24			
Histology (squamous vs. nonsquamous)	2.30	1.21–4.37	0.011	2.40	1.17–4.93	**0.017**
PD-L1 TPS (other vs. ≥1%)	1.02	0.54–1.93	0.95			
Stage (vs. IIIA)						
IIIB	1.23	0.55–2.41	0.55			
IIIC	1.59	0.54–4.71	0.40			
LIPI groups (vs. Good)						
Intermediate	1.20	0.58–2.47	0.63	1.04	0.46–2.03	0.92
Poor	1.15	0.40–3.31	0.80	3.56	1.08–11.7	**0.037**
Number of durvalumab infusions (continuous)	0.87	0.83–0.91	<0.0001	0.86	0.82–0.90	**<0.0001**
Percentage durvalumab infusion after 15:00 h (≥20% vs. <20%)	2.43	1.11–5.34	0.027	2.54	1.03–5.67	**0.047**

Overall survival	Univariable Analysis			Multivariable Analysis^a^		
Variable	HR	95% CI	*p*	HR	95% CI	*p*
Age (≥65 vs. <65 years)	1.11	0.41–1.97	0.78			
Sex (male vs. female)	0.91	0.36–2.24	0.83			
ECOG PS (≥1 vs. 0)	1.40	0.66–2.97	0.38			
Histology (squamous vs. nonsquamous)	3.28	1.56–6.93	0.002	1.10	1.34–6.64	**0.007**
PD-L1 TPS (other vs. ≥1%)	1.04	0.50–2.16	0.93			
Stage (vs. IIIA)						
IIIB	1.25	0.80–2.72	0.58			
IIIC	1.48	0.43–5.13	0.54			
LIPI groups (vs. Good)						
Intermediate	1.17	0.52–2.63	0.71			
Poor	1.61	0.55–4.73	0.39			
Number of durvalumab infusions (continuous)	0.88	0.84–0.92	<0.0001	0.88	0.84–0.92	**<0.0001**
Percentage durvalumab infusion after 15:00 h (≥20% vs. <20%)	1.80	0.73–4.42	0.20	1.28	0.51–3.25	0.60

CI, confidence interval; ECOG, Eastern Cooperative Oncology Group; HR, hazard ratio; LIPI, lung immune prognostic index; PS, performance status.

Bold text indicates statistical significance.

a Backward stepwise multivariable Cox regression model was used.

After PSM, a total of 30 patients were included, with all confounding factors balanced between the two groups (Supplementary Table 2). Among these PSM patients, those who received at least 20% of their durvalumab infusions after 3 PM had a significantly shorter PFS and OS compared with those who did not (median PFS: 9.7 mo versus NA; HR, 4.64; 95% CI: 1.95–11.04; *p* < 0.001 [Fig. 1*C*]; median OS: 18.3 mo versus NA; HR, 5.33; 95% CI: 2.17–13.06; *p* < 0.001 [Fig. 1*D*]).

In a sensitivity analysis in which the cutoff time was adjusted to 2:30 PM, a backward stepwise multivariable analysis revealed that patients receiving at least 20% of durvalumab infusions after 2:30 PM were associated with worse PFS (HR, 3.24; 95% CI: 1.45–7.22; *p* = 0.0004) but did not exhibit significantly worse OS (HR, 2.01; 95% CI: 0.88–4.61; *p* = 0.090). These findings are detailed in Supplementary Table 3 and Supplementary Figure 2.

## Discussion

In our study, patients with at least 20% of their durvalumab infusions after 3 PM had a significantly shorter PFS. In addition, both multivariate analysis and PSM analysis revealed that receiving at least 20% of durvalumab infusions after 3 PM was statistically associated with worse PFS; however, this association was not statistically significant for OS. When the cutoff time was adjusted to 2:30 PM, a similar difference was observed.

The association between the response to ICI and the timing of ICI infusions was recently reported for various cancer types (Supplementary Table 1).^6^^,^^13^^,^^14^ Seven retrospective studies of patients with melanoma, metastatic renal cell carcinoma, and esophageal squamous cell carcinoma exhibited worse survival outcomes when most of the ICI infusions were administered in the evening.^6^^,^^14^ Moreover, five retrospective studies identified a similar association in metastatic NSCLC treated with ICI.^7^, ^8^, ^9^^,^^15^^,^^16^ In line with these findings, the present study revealed that frequent evening administration of ICI was associated with worse survival outcomes in patients with locally advanced NSCLC. The present study is the first to investigate this potential association in patients with nonmetastatic cancers. Adjusting the timing of ICI infusions to avoid evening administration might be a straightforward, cost-free step to improving the clinical outcomes of ICI therapy, including that for locally advanced NSCLC.

Preclinical research has identified a diurnal oscillation in the number of circulating programmed cell death protein 1 (PD-1)–expressing tumor-associated macrophages, dendritic cells, and CD8+ T cells.^3^^,^^14^^,^^17^

Increased CD8+ T cells in the patient’s blood were observed when vaccinations were performed in the morning compared with the afternoon.^3^ Moreover, ICI's efficacy was enhanced when the drug was administered at the time of day when PD-1 expression on tumor-associated macrophages increased.^17^ Furthermore, circadian variations in the number and composition of T cells are differentially regulated through the release of cortisol and catecholamines.^18^ These findings suggest that lower T cell numbers and PD-1 expression levels in the afternoon might lead to reduced ICI efficacy.^14^ Clinical trials of chronomodulated therapy reported the effect of a circadian schedule for adoptive cytokine therapy two decades ago.^19^^,^^20^ Larger prospective studies are warranted to evaluate the impact of chronomodulation on ICI efficacy in cancer treatment.

The limitations of our study include its retrospective design. In addition, the study was conducted at a single center, thus, possibly limiting the generalizability of its findings. The relatively small sample size might have hampered our efforts to detect differences in survival outcomes by means of multivariable analysis, and translational data supporting the clinical results were lacking. In addition, although the choice of a 20% cutoff for the proportion of infusions was in line with previous studies, there was no clear consensus about the definition of cutoff for the time and proportion. In addition, in most Japanese hospital organizations, cancer treatments are primarily scheduled before 3 PM. Infusions might be administered after this time for organizational reasons or because of treatment delays necessitated by the clinical situation, such as the need for additional examinations. These delays might constitute an important unquantifiable contributing factor, although our PFS findings were consistent on both univariable, multivariable, and PSM analysis.

In conclusion, the time of day of durvalumab infusions may influence survival outcomes in patients with locally advanced NSCLC. Prospective trials are needed to verify the association between the timing of ICI infusions and clinical outcomes.

## CRediT Authorship Contribution Statement

Tsuyoshi Hirata: Resources, Investigation, Writing - Original Draft.

Yuji Uehara: Conceptualization, Software, Formal Analysis, Methodology, Resources, Investigation, Writing - Original Draft, Supervision.

Taiki Hakozaki: Supervision, Writing - Review & Editing.

Takayuki Kobayashi: Resources, Writing - Review & Editing.

Yuto Terashima: Resources, Writing - Review & Editing.

Kageaki Watanabe: Supervision, Writing - Review & Editing.

Makiko Yomota: Writing - Review & Editing.

Yukio Hosomi: Project administration, Writing - Review & Editing.

## Disclosures

Dr. Hakozaki has received payment for speakers’ bureaus from Chugai Pharmaceutical outside the submitted work. Dr. Watanabe has received honoraria as a speaker from AstraZeneca, Chugai Pharmaceutical, MSD, Boehringer Ingelheim, Takeda Pharmaceutical, Bristol Myers Squibb, Merck Biopharma, and Ono Pharmaceutical outside the submitted work. Dr. Yomota has received honoraria for speakers’ bureaus from Chugai Pharmaceutical, Ono Pharmaceutical, AstraZeneca, Taiho Pharmaceutical, Takeda Pharmaceutical, Boehringer Ingelheim, and Pfizer outside the submitted work. Dr. Hosomi has received personal fees from AstraZeneca, Bristol Myers Squibb, Chugai Pharmaceutical, Eisai, Eli Lilly, Kyowakirin, Nippon Kayaku, Novartis Ono Pharmaceutical, Takeda, and Taiho Pharmaceutical outside the submitted work. The remaining authors declare no conflict of interest.
