# Supplementary material for: Brief Report: Clinical Outcomes by Infusion Timing of Immune Checkpoint Inhibitors in Patients With Locally Advanced NSCLC
Source: JTO Clin Res Rep. 2024 Mar 5;5(4):100659. doi: 10.1016/j.jtocrr.2024.100659 (PMC11001639; doi:10.1016/j.jtocrr.2024.100659)
Supplement: Supplementary Tables 1 and 2 [file mmc1.docx]

**Supplementary Figure 1. Progression-free survival (A) and overall survival (B) according to histological subtypes (squamous cell carcinoma versus non-squamous cell carcinoma)
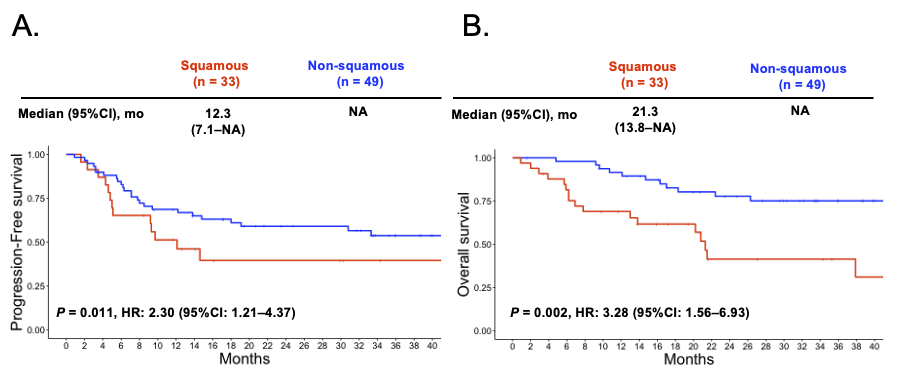
**

**Supplementary Figure 2. Progression-free survival (A) and overall survival (B) according to 20% threshold in percentage of ICI infusions received after 14:30h**

**
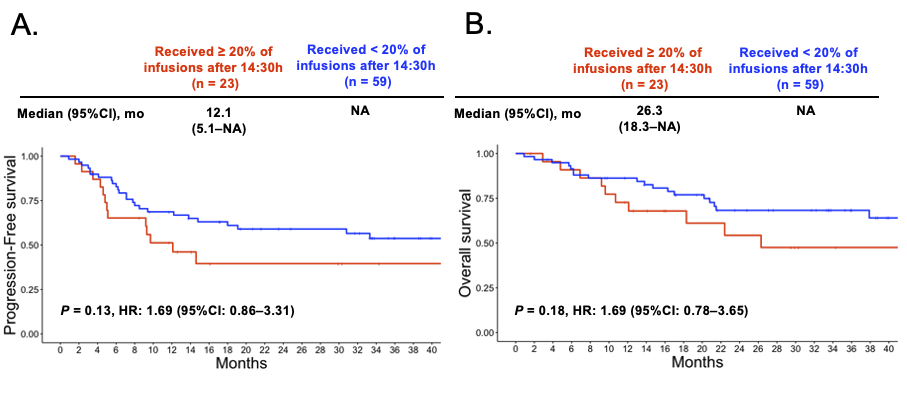
**

**Supplementary Table 1. Previous studies investigating the association between the timing of immune checkpoint inhibitor administration during the day and treatment efficacy**

| **Reference** | **Cancer types** | **Stage** | **ICI treatment** | **Line of treatment** | **Total number of patients** | **Time cutoff for evening** | **Key result** |
| --- | --- | --- | --- | --- | --- | --- | --- |
| Single center analysis by Qian, et al. 2021 ^1^ | Melanoma | Ⅳ | Pembrolizumab, Ipilimumab, Nivolumab, Dual ICIs | Any line | 146 | 16:30 | Having at least 20% of infusions in the evening was significantly associated with shorter OS (median: 4.8 years vs. NA; HR: 2.04 [1.04–4.00], *P* = 0.038]). |
| Single center analysis by  Gonçalves, et al. 2023 ^2^ | Melanoma | Ⅳ | Nivolumab, Pembrolizumab, Nivolumab + Ipilimumab | Any line | 73 | 14:00 | OS was significantly longer in the morning group: a median of 38.1 versus 14.2 months in the afternoon group, with a HR of 0.45 (95% CI 0.23–0.86; *P* < 0.01). |
| Single center analysis by  Yeung, et al. 2023 ^3^ | Melanoma | Ⅳ | Nivolumab, Pembrolizumab, Nivolumab + Ipilimumab | Any line | 121 | 13:00 | OS was significantly longer in the morning group: a median of 24.9 versus 5.5 months in the afternoon group. |
| Single center analysis by  Rousseau, et al. 2023 ^4^ | NSCLC | Ⅳ | Nivolumab, Pembrolizumab, Atezolizumab | Any line | 180 | 16:30 | Having at least 20% of infusions in the evening was significantly associated with shorter PFS (median: 4.9 vs. 9.4 months, *P* = 0.020]). |
| Multicenter analysis by  Cortellini, et al. 2022 ^5^ | NSCLC | Ⅳ | Pembrolizumab | First line | 180 | 16:30 | Having at least 20% of infusions in the evening was associated with shorter PFS (median: 6.6 vs. 19.7 months, *P* = 0.056]) in the propensity score matching. |
| Single center analysis by  Karaboué, et al.2022 ^6^ | NSCLC | Ⅳ | Nivolumab | Second or later line | 95 | 12:55 | PFS was significantly longer in the morning group: a median of 11.3 versus 3.1 months in the afternoon group (*P* < 0.001).  OS was significantly longer in the morning group: a median of 34.2 versus 9.6 months in the afternoon group (*P* < 0.001). |
| Multicenter analysis by  Barrios, et al. 2022^7^ | NSCLC | Ⅳ | Nivolumab, Pembrolizumab, Atezolizumab | Any line | 129 | 16:00 | OS was longer in the morning group: a median of 14 versus 4.9 months in the afternoon group. |
| Single center analysis by  Vilatla et al. 2021^8^ | NSCLC | Ⅳ | Nivolumab, Pembrolizumab, Atezolizumab | Any line | 197 | 12:00 | PFS was significantly longer in the morning group: a median of 6.5 versus 3.2 months in the afternoon group, with a HR of 0.42 (95% CI 0.28–0.64; *P* < 0.01). |
| **Single center analysis by Hirata, et al. 2023 (our study)** | **NSCLC** | **Ⅲ** | **Durvalumab** | **Consolidation therapy** | **82** | **15:00** | **Having at least 20% of infusions in the evening was significantly associated with shorter PFS (median: 7.4 months vs. NA; HR: 2.43 [1.11–5.34], *P* = 0.027]).** |
| Single center analysis by  Dizman, et al. 2023 ^9^ | Renal cell carcinoma | Ⅳ | Nivolumab, ipilimumab | First or second line | 135 | 16:30 | Having at least 20% of infusions in the evening was significantly associated with shorter TTF (median: 4.6 vs. 9.5 months; HR: 1.69 [1.06–2.70], *P* = 0.026]) in the multivariable analysis. |
| Single center analysis by  Fernandez-Mañas, et al. 2023 ^10^ | Renal cell carcinoma | Ⅳ | Nivolumab, Ipilimumab,  Nivolumab + Ipilimumab | Any line | 104 | 16:30 | Having at least 20% of infusions in the evening was significantly associated with shorter TOT (median: 4.3 vs. 9.0 months; HR: 2.5, *P* = 0.013]). |
| Single center analysis  by Patel et al. 2023 ^11^ | Renal cell carcinoma |  | Nivolumab, Ipilimumab,  Nivolumab + Ipilimumab | Any line | 201 | 13:00 | OS was significantly longer in the morning group: a median of 59 versus 34 months in the afternoon group, with a HR of 0.51 (*P* = 0.017). |
| Multicenter analysis  by Ortego et al. 2023 ^12^ | Urothelial cancer | Ⅳ | Nivolumab, Pembrolizumab, Atezolizumab | Any line | 88 | 16:30 | PFS was significantly longer in the morning group: a median of 11.4 versus 3.6 months in the afternoon group, with a HR of 0.38 (95% CI 0.21–0.65; *P* < 0.001). |
| Single center analysis by  Nomura, et al. 2023 ^13^ | Esophageal squamous cell carcinoma | Ⅳ | Nivolumab | Any line | 62 | 13:00 | PFS of patients who received the majority of nivolumab infusions before 13:00 during the first 3 months were significantly superior to those who received it after 13:00 ( HR: 0.40 [0.22–0.71], *P* = 0.002]). |

Abbreviations: CI, confidence interval; HR, hazard ratio; Not available, NA; OS, overall survival; PFS, progression-free survival; TOT, time on treatment; TTF, time to treatment failure.

**References for Supplementary Table 1**

1. Qian DC, Kleber T, Brammer B, et al. Effect of immunotherapy time-of-day infusion on overall survival among patients with advanced melanoma in the USA (MEMOIR): a propensity score-matched analysis of a single-centre, longitudinal study. *Lancet Oncol*. 2021;22(12):1777-1786. doi:10.1016/S1470-2045(21)00546-5

2. Gonçalves L, Gonçalves D, Esteban-Casanelles T, et al. Immunotherapy around the Clock: Impact of Infusion Timing on Stage IV Melanoma Outcomes. *Cells*. 2023;12(16):2068. doi:10.3390/cells12162068

3. Yeung C, Kartolo A, Tong J, Hopman W, Baetz T. Association of circadian timing of initial infusions of immune checkpoint inhibitors with survival in advanced melanoma. *Immunotherapy*. 2023;15(11):819-826. doi:10.2217/imt-2022-0139

4. Rousseau A, Tagliamento M, Auclin E, et al. Clinical outcomes by infusion timing of immune checkpoint inhibitors in patients with advanced non-small cell lung cancer. *Eur J Cancer*. 2023;182:107-114. doi:10.1016/j.ejca.2023.01.007

5. Cortellini A, Barrichello APC, Alessi JV, et al. A multicentre study of pembrolizumab time-of-day infusion patterns and clinical outcomes in non-small-cell lung cancer: too soon to promote morning infusions. *Ann Oncol Off J Eur Soc Med Oncol*. 2022;33(11):1202-1204. doi:10.1016/j.annonc.2022.07.1851

6. Karaboué A, Collon T, Pavese I, et al. Time-Dependent Efficacy of Checkpoint Inhibitor Nivolumab: Results from a Pilot Study in Patients with Metastatic Non-Small-Cell Lung Cancer. *Cancers*. 2022;14(4):896. doi:10.3390/cancers14040896

7. Barrios CH, Montella TC, Ferreira CGM, et al. Time-of-day infusion of immunotherapy may impact outcomes in advanced non-small cell lung cancer patients (NSCLC). *J Clin Oncol*. 2022;40(16_suppl):e21126-e21126. doi:10.1200/JCO.2022.40.16_suppl.e21126

8. Vilalta A, Arasanz H, Rodriguez-Remirez M, et al. 967P The time of anti-PD-1 infusion improves survival outcomes by fasting conditions simulation in non-small cell lung cancer. *Ann Oncol*. 2021;32:S835. doi:10.1016/j.annonc.2021.08.1352

9. Dizman N, Govindarajan A, Zengin ZB, et al. Association Between Time-of-Day of Immune Checkpoint Blockade Administration and Outcomes in Metastatic Renal Cell Carcinoma. *Clin Genitourin Cancer*. 2023;21(5):530-536. doi:10.1016/j.clgc.2023.06.004

10. Fernandez-Mañas L, Gonzalez Aguado L, Aversa C, et al. Does the time-of-day administration of immune checkpoint inhibitors affect efficacy in patients with metastatic renal cell carcinoma? A single-center study. *J Clin Oncol*. 2023;41(6_suppl):681-681. doi:10.1200/JCO.2023.41.6_suppl.681

11. Patel J, Draper A, Woo Y, et al. 848 Impact of immunotherapy time-of-day infusion on overall survival in patients with metastatic renal cell carcinoma. *J Immunother Cancer*. 2022;10(Suppl 2). doi:10.1136/jitc-2022-SITC2022.0848

12. Ortego I, Molina-Cerrillo J, Pinto A, et al. Time-of-day infusion of immunotherapy in metastatic urothelial cancer (mUC): Should it be considered to improve survival outcomes? *J Clin Oncol*. 2022;40(16_suppl):e16541-e16541. doi:10.1200/JCO.2022.40.16_suppl.e16541

13. Nomura M, Hosokai T, Tamaoki M, Yokoyama A, Matsumoto S, Muto M. Timing of the infusion of nivolumab for patients with recurrent or metastatic squamous cell carcinoma of the esophagus influences its efficacy. *Esophagus*. Published online April 24, 2023:1-10. doi:10.1007/s10388-023-01006-y

**Supplementary Table 2. Patient characteristics after PSM**

|  | Whole population  (N = 30) | Percentage infusion after 15:00h <20%  (n = 25) | Percentage infusion after 15:00h >20%  (n = 5) | *P* |
| --- | --- | --- | --- | --- |
| Age (median, IQR), n (%)  ≤65 year  >65 year | 10 (33)  20 (67) | 9 (36)  16 (64) | 1 (20)  4 (80) | 0.86 |
| Sex, n (%)  Male  Female | 29 (97)  1 (3) | 24 (96)  1 (4) | 5 (100)  0 | 1.00 |
| ECOG-PS, n (%)  0  1  ≥2 | 16 (53)  12 (40)  2 (7) | 14 (55)  10 (40)  1 (4) | 2 (40)  2 (40)  1 (20) | 0.41 |
| Smoking status, n (%)  Current or former  Nonsmoker | 30 (100)  0 | 25 (100)  0 | 5 (100)  0 | 1.00 |
| Histological subtypes, n (%)  Non-squamous cell carcinoma  Squamous cell carcinoma | 20 (67)  10 (33) | 17 (68)  8 (32) | 3 (60)  2 (40) | 1.00 |
| PD-L1 expression, n (%)  ≥1%  Other | 15 (50)  15 (50) | 12 (48)  13 (52) | 3 (60)  2 (40) | 1.00 |
| AJCC eighth edition, overall stage, n (%)  IIIA  IIIB  IIIC | 16 (53)  9 (30)  5 (17) | 14 (56)  7 (28)  4 (16) | 2 (40)  2 (40)  1 (20) | 0.67 |
| LIPI groups^a^  Good  Intermediate  Poor | 8 (27)  16 (53)  6 (20) | 7 (28)  13 (52)  5 (20) | 1 (20)  3 (60)  1 (20) | 0.80 |
| Number of durvalumab infusions (median, IQR) | 14 (6–24) | 17 (8–24) | 12 (6–19) | 0.063 |

^a^ Good, intermediate, and poor LIPI is based on the pretreatment lab values following cutoffs: dNLR ≤3 and LDH ≤ ULN, dNLR >3 or LDH > ULN, and dNLR >3 and LDH > ULN.

**Abbreviations**: AJCC, American Joint Committee on Cancer; ECOG, Eastern Cooperative Oncology Group; IQR, interquartile range; LDH, lactate dehydrogenase; LIPI, Lung Immune Prognostic Index; Neutrophil-to-lymphocyte ratio, NLR; PS, performance status; PSM, propensity score-matching; PD-L1, programmed cell death ligand ; ULN, upper limit normal.

**Supplementary Table 3. Stepwise multivariable Cox model analysis for progression-free survival and overall survival (cutoff: 14:30h)**

| **Progression-free Survival** | **Univariable analysis** | | | **Multivariable analysis^a^** | | |
| --- | --- | --- | --- | --- | --- | --- |
| **Variable** | **HR** | **95% CI** | ***P*** | **HR** | **95% CI** | ***P*** |
| Age (≥65 vs. <65 years) | 1.55 | 0.81–2.97 | 0.19 |  |  |  |
| Sex (Male vs. Female) | 0.93 | 0.41–2.14 | 0.88 |  |  |  |
| ECOG PS (≥1 vs. 0) | 1.48 | 0.77–2.84 | 0.24 |  |  |  |
| Histology (Squamous vs. Non-squamous) | 2.30 | 1.21–4.37 | 0.011 | 3.21 | 1.47–7.00 | **0.017** |
| PD-L1 TPS (Other vs. ≥1%) | 1.02 | 0.54–1.93 | 0.95 |  |  |  |
| Stage (vs. IIIA)  IIIB  IIIC | 1.23  1.59 | 0.55–2.41  0.54–4.71 | 0.55  0.40 |  |  |  |
| LIPI groups (vs. Good)  Intermediate  Poor | 1.20  1.15 | 0.58–2.47  0.40–3.31 | 0.63  0.80 | 1.11  5.40 | 0.52–2.39  1.57–18.6 | 0.92  0.037 |
| Number of durvalumab infusions (continuous) | 0.87 | 0.83–0.91 | <0.0001 | 0.85 | 0.81–0.89 | **<0.0001** |
| Percentage durvalumab infusion after 14:30h (≥20% vs. <20%) | 1.69 | 0.86–3.31 | 0.13 | 3.24 | 1.45–7.22 | **0.0004** |
| **Overall survival** | **Univariable analysis** | | | **Multivariable analysis^a^** | | |
| **Variable** | **HR** | **95% CI** | ***P*** | **HR** | **95% CI** | ***P*** |
| Age (≥65 vs. <65 years) | 1.11 | 0.41–1.97 | 0.78 |  |  |  |
| Sex (Male vs. Female) | 0.91 | 0.36–2.24 | 0.83 |  |  |  |
| ECOG PS (≥1 vs. 0) | 1.40 | 0.66–2.97 | 0.38 |  |  |  |
| Histology (Squamous vs. Non-squamous) | 3.28 | 1.56–6.93 | 0.002 | 3.82 | 1.66–8.79 | **0.007** |
| PD-L1 TPS (Other vs. ≥1%) | 1.04 | 0.50–2.16 | 0.93 |  |  |  |
| Stage (vs. IIIA)  IIIB  IIIC | 1.25  1.48 | 0.80–2.72  0.43–5.13 | 0.58  0.54 | 2.11  4.17 | 0.88–5.03  0.96–18.0 | 0.093  0.056 |
| LIPI groups (vs. Good)  Intermediate  Poor | 1.17  1.61 | 0.52–2.63  0.55–4.73 | 0.71  0.39 |  |  |  |
| Number of durvalumab infusions (continuous) | 0.88 | 0.84–0.92 | <0.0001 | 0.86 | 0.81–0.91 | **<0.0001** |
| Percentage durvalumab infusion after 14:30h (≥20% vs. <20%) | 1.69 | 0.78–3.65 | 0.18 | 2.01 | 0.88–4.61 | 0.090 |

**^a^** backward stepwise multivariable Cox regression model was used

**Abbreviations**: CI, confidence interval; ECOG, Eastern Cooperative Oncology Group; HR, hazard ratio; LIPI, Lung Immune Prognostic Index; PS, performance status.
